# Knowledge of recommended antibiotic treatments for community-acquired infections in general medical practice: a cross-sectional study in *Occitanie* region, France

**DOI:** 10.1038/s41598-023-43809-0

**Published:** 2023-10-12

**Authors:** Alessio Strazzulla, Manuel Ballarin, Maria Concetta Postorino, Raphaël Lee, Pierre Leroy, Bernard Castan, Sylvain Diamantis

**Affiliations:** 1 Internal Medicine Unit, Groupe Hospitalier Sud Ile de France, Melun, France; 2 Infectious Diseases Unit, Groupe Hospitalier Sud Ile de France, Melun, France; 3 Public Health Unit, Groupe Hospitalier Sud Ile de France, Melun, France; 4 Infectious Diseases Unit, Centre Hospitalier de Périgueux, Périgueux, France; 5 Research Unit “DYNAMIC”, Université Paris-Est Créteil, Créteil, France

**Keywords:** Bacterial infection, Antibiotics

## Abstract

To assess and analyse the knowledge of recommended antibiotic treatments, focusing on the appropriate drugs and treatment durations for the most common community-acquired infections in general medical practice in Occitanie region, France. A web-based survey was conducted over a 3-month period, from October, 2018 to January, 2019. All participants answered directly through the online platform. For the analysis of overtreatment risk, a score based system was adopted and two scores were produced: the duration score and the treatment score. 413 general practitioners completed the survey. The overall rate of concordance with guidelines in terms of both drug choice and treatment length was 2974/4956 (60%) answers. Diseases with at least 70% good answers included cystitis, group A streptococcal pharyngitis, and bacterial superficial skin infections. Diseases with fewer than 50% good answers included pyelonephritis, dog bite wounds, and community-acquired pneumonia in patients aged ≥ 65 years. Factors associated with the risk of overtreatment were age > 40 years, country setting and hospital employment. Knowledge of treatment durations is satisfactory with 60% of recommendations being met. However, varying levels were observed according to different diseases. This study highlighted a very high rate of adherence when recommendations were clear. In contrast, low levels of adherence were observed when recommendations were ambiguous or when conflicting guidelines existed.

## Introduction

Antibiotic over-prescription is a significant public health issue. Indeed, excessive antibiotic use escalates public expenditure and propels the spread of antibiotic-resistant bacteria^1^. In terms of public health, it is advisable to reduce the treatment duration to minimize the risk of selecting and proliferating resistant bacteria. Consequently, antibiotic stewardship programs focused on reducing antibiotic treatment durations. In alignment with this objective, the French Society of Infectious Diseases (*Societé de Pathologie Infectieuse de Langue Française* or SPILF) released guidelines in 2017 specifically aimed at reducing antibiotic treatment durations^2^. These guidelines were updated in 2021^3^.

Over the past two decades, antibiotic consumption in France has followed a three-phase trend. Between 2000 and 2004, there was a notable decrease of 18.9%, coinciding with the national campaign “Antibiotics are not systematic”. This was followed by a period of stability from 2005 to 2010. However, from 2011 to 2020, there was an increase in antibiotic consumption by 8.6%^4^. Currently, the rate stands at 32.1 daily defined dose (DDD)/1000 habitants/day in France, compared to the European Union’s 22.4 DDD/1000 habitants/day. A staggering 93% of these prescriptions come from the outpatient sector, with general practitioners (GP) accounting for 71% of them. Primary drivers for antibiotic prescriptions include ear-nose-throat (ENT) infections (42%), lower respiratory tract infections (25%) and urinary tract infections (8%)^1^. These statistics place France among the top antibiotic consumers in Europe^5^.

Alterations in antibiotic prescription behaviours and emphasizing the appropriate use of antibiotics are fundamental aspects of antibiotic stewardship programs. The appropriate use of antibiotics refers to conditions that ensure an optimal benefit-to-risk ratio when administering a specific drug to an individual^6^. In practical terms, this entails a judicious antibiotic prescription, with the appropriate drug, for a set duration, and adhering to a specified dosage regimen. Nonetheless, few studies have assessed the knowledge level concerning the recommended antibiotic treatments in terms of appropriate molecule and treatment duration for community-acquired infections in general practice.

The primary objective of this study was to evaluate and analyse the knowledge level of recommended antibiotic treatments in terms of appropriate drug and treatment duration for the most common community-acquired infections encountered in general practice.

The secondary objectives included:

i. Identifying the conditions most often associated with a treatment duration exceeding guideline recommendations.
ii. Determining the factors associated with extended antibiotic treatment.

## Materials and methods

### Study design

A web-based survey was conducted over a span of three months, from October 15th, 2018, to January 15th, 2019. GPs and general medicine residents from the *Occitanie* region in France had free access to the survey on the WebQuest® online platform. The survey was distributed via web mails, scientific journals, and social media. Collaborative entities included: the regional union of health care professionals of *Occitanie*, the network of general medicine residents from *Toulouse-Purpan* University’s Medical Faculty, the group of referents in extra-hospital infective surveillance, and the young infectious diseases specialists. To maximize GP participation, the survey was also promoted on social media platforms. All respondents submitted their answers directly through the online platform. Submissions were fully anonymized. The survey in French language is available on reasonable request to the corresponding author. Data from all respondents were analysed without exclusions.

### Survey

The survey was designed by two infectious diseases specialists, members of the board of the French Society of Infectious Diseases and authors of the French guidelines on antibiotic treatment duration^3^. The basis for the questionnaire was the study by Cecilia Costa, validated at Marseille University on May 23rd, 2018^7^. The survey was anonymous and structured in two sections : (i) Demographic and professional characteristics of the respondent [including gender, age, setting (urban, rural, or both), and workplace (clinic, hospital medical unit, hospital emergency unit, nursing home, or independent practice)]; (ii) Clinical cases covering 12 distinct conditions: group A streptococcal pharyngitis (GASP), bacterial maxillary sinusitis, bacterial frontal sinusitis, congestive otitis in children, cystitis in women, pyelonephritis in women, urinary tract infections (UTI) in men, bacterial superficial skin infections, dog bite wounds, community-acquired pneumonia (CAP) in patients aged ≥ 65 years, and mild exacerbation of chronic obstructive pulmonary disease (COPD). Respondents were presented with open-ended questions regarding empirical antibiotic treatment and multiple-choice questions on treatment duration. The gold standard for treatment was established using the 2017 French national guidelines endorsed by the SPILF^2^. Exceptionally, for COPD exacerbation, guidelines from both the SPILF and the French Agency for the Sanitary Safety of Health Products (*Agence Française de Sécurité Sanitaire des Produits de Santé*or AFSSAPS) were referenced due to their divergent recommendations (antibiotic treatment as per SPILF vs no antibiotic treatment as per AFSSAPS)^8^.

To analyse the overtreatment risk, a scoring system was implemented, resulting in two scores: the duration score and the treatment score. In practice, answers to the twenty-four questions (twelve on the choice of molecules and twelve on antibiotic treatment duration) submitted by each respondent were analysed. Responses aligning with the durations and/or types of drugs suggested by the national guidelines were deemed correct. For both the duration and treatment scores, a value of 0 points was allocated for each correct answer, while 1 point was allocated for each incorrect answer. The cumulative score was then calculated. Finally, prescribers with a duration or treatment score exceeding the 75th percentile were identified.

### Statistical analysis

The required number of participants was calculated based on a standard p-value of 0.05, a precision of 0.05, and an arbitrarily designed expected proportion (p) of physicians in agreement with the measures set at 20%. Using the formula n = 1.962 × p (1–p), the minimal number of physicians necessary to yield a statistically significant outcome was determined to be 245.

Diseases were categorized into three groups based on the percentage of correct answers: (i) diseases with a low level of knowledge (≤ 50% of correct answers); (ii) diseases with a moderate level of knowledge (51–69% of correct answers); (iii) diseases with a high level of knowledge (≥ 70% of correct answers).

For statistical analysis, we used *Excel 2016®* software and the *pvalue.io©* website. Descriptive analysis displayed data as either mean values or observed number of events. Student’s *t* test was used for inferential analysis. The following analysis were performed : (i) descriptive analysis of the characteristics of each practitioner ; (ii) descriptive analysis of the responses to all 12 questions; (iii) comparison between the characteristics of practitioners with a duration score exceeding the 75th percentile and those with a duration score equal to or below the75th percentile; (iv) comparison between the characteristics of practitioners with a treatment score above the 75th percentile and those with a duration score equal to or below the 75th percentile.

### Ethical considerations

This study was conducted in accordance with the Declaration of Helsinki, as well as other national and institutional standards^9,10^. Methodological and ethical considerations received approval from the Medical Department of Pierre and Marie Curie University in Paris, France, on January 25th, 2018. An electronic consent form was signed by each respondent.

## Results

A total of 413 physicians responded to the survey. The characteristics of the participants are detailed in Table 1. The overall adherence to the guidelines, in terms of both appropriate molecule and treatment duration was 2974/4956 (60%) responses. Tables 2 and 3 summarize the results of the survey.

**Table 1:** Characteristics of the population.

Characteristics	Physicians
	n = 413
Demographical parameters	
Median age, years [IQR]	31 [7]
Male gender, n [%]	123 [30]
Female gender, n [%]	290 [70]
Location	
City, n [%]	179 [43]
City and country*, n [%]	116 [28]
Country, n [%]	87 [21]
Missing data, n [%]	31 [8]
Workplaces**	
Clinic, n [%]	31 [7]
Hospital medical unit, n [%]	25 [6]
Hospital emergency unit, n [%]	24 [6]
Nursing home, n [%]	7 [2]
Private practice, n [%]	326 [79]

*IQR* interquartile range.

*It means that practitioners worked in two different locations, **For practitioners with two or more workplaces, only the main workplace was considered. The main work place was defined as the place where it spent > 50% of its worktime.

**Table 2:** Prescribed and recommended treatments.

Disease		Recommended molecules*	Prescribed molecules		Adapted molecule	Recommended treatment lenght or rtl*	Treatment lenght		Mean treatment lenght	Treatment lenght difference	Adapted molecule + treatment lenght
		First line molecules	drugs	n/tot (%)	n/tot (%)	Days	Days (%)		Mean (SD)	Days	n/tot (%)
Ear-nose-throat	GASP^1^	Amoxicillin	Amoxicillin	407/413 (99)	407/413 (99)	6	< RTL	59/413 (14)	5.9 (0.5)	– 0.1	320/413 (77)
			Amoxicillin/clavulanic acid	5/413 (1)			= RTL	320/413 (77)			
			Other molecules	1/413 (0)			> RTL	34/413 (8)			
	Bacterial maxillary sinusitis	Amoxicillin	Amoxicillin	297/413 (72)	297/413 (72)	7			6.7 (1.0)	– 0.3	223/413 (60)
			Amoxicillin/clavulanic acid	49/413 (12)			< RTL	78/413 (21)			
			Pristinamycin	21/413 (5)			= RTL	264/413 (70)			
			Other molecules	9/413 (2)			> RTL	32/413 (9)			
			No treatement	37/413 (9)							
	Bacterial frontal sinusitis	Amoxicillin/clavulanic acid	Amoxicillin/clavulanic acid	328/413 (79)	328/413 (79)	7			7.1 (1.0)	+ 0.1	249/413 (61)
			Amoxicillin	56/413 (14)			< RTL	57/413 (14)			
			Macrolids and streptogramins	10/413 (2%)			= RTL	283/413 (70)			
			Other molecules	13/413 (3)			> RTL	67/413 (16)			
			No treatement	6/413 (1)							
	Congestive otitis (paediatric)	No treatment	Amoxicillin	118/413 (29)	289/413 (70)	0			5.8 (1.1)	+ 5.8	289/413 (70)
			Other molecules	6/413 (1)			> RTL	124/413 (30)			
			No treatment	289/413 (70)							
Urinary	Cystitis (women)	Fosfomycin	Fosfomycin	397/413 (96)	397/413 (97)	1	< RTL	3/413 (1)	1.1 (1.0)	– 0.1	397/413 (97)
			Other molecules	13/413 (3)			= RTL	397/413 (96)			
			No treatment	3/413 (1)			> RTL	12/413 (3)			
	Pyelonephritis (women)	Fluoroquinolones or 3GC (intramuscular)	Fluoroquinolones	311/413 (76)	367/413 (90)	7			8.5 (2.4)	+ 1.5	182/413 (48%)
			3GC (intramuscular)	56/413 (14)			< RTL	24/413 (6)			
			Amoxicillin	22/413 (5)			= RTL	194/413 (51)			
			3GC (oral)	14/413 (3)			> RTL	159/413 (42)			
			Other molecules	10/413 (2)							
	UTI (men)	Fluoroquinolones**	Fluoroquinolones	361/413 (88)	361/413 (88)	14			NA	NA	224/413 (54)
			3GC (intramuscular)	32/413 (8)			< RTL	53/413 (15)			
			TMP-SMX	10/413 (2)			= RTL	224/413 (62)			
			3GC (oral)	5/413 (1)			> RTL	69/413 (19)			
			Other molecules	5/413 (1)							
Skin	Bacterial superficial skin infections	Amoxicillin	Amoxicillin	347/413 (84)	347/413 (84)	7			9.5 (2.1)	+ 2.5	308/413 (75)
			Amoxicillin/clavulanic acid	33/413 (8)			< RTL	9/413 (2)			
			Pristinamycin	23/413 (6)			= RTL	359/413 (87)			
			Cloxacillin	8/413 (2)			> RTL	45/413 (11)			
			Fusidic acid	2/413 (0)							
	Dog bite wounds	Amoxicillin/clavulanic acid	Amoxicillin/clavulanic acid	359/413 (87)	359/413 (87)	5			6.2 (1.7)	+ 1.2	166/413 (41)
			Pristinamycin	24/413 (6)			< RTL	17/413 (4)			
			Amoxicillin	11/413 (3)			= RTL	173/413 (43)			
			Doxycyclin	5/413 (1)			> RTL	208/413*** (52)			
			No treatment	14/413 (3)							
Pulmonary	Community acquired pneumonia (≥ 65 year old)	Amoxicillin/clavulanic acid or Fluoroquinolones or 3GC (intramuscular)	Amoxicillin	229/413 (56)	179/413 (43)	7			8.0 (1.8)	+ 1.0	112/413 (27)
			Amoxicillin/clavulanic acid	170/413 (41)			< RTL	19/413 (5)			
			Fluoroquinolones	5/413 (1)			= RTL	241/413 (58)			
			3GC (intramuscular)	4/413 (1)			> RTL	152/413 (37)			
			Macrolids and streptogramins	5/413 (1)							
	COPD exacerbations (mild)	Amoxicillin, amoxicillin/clavulanic acid, macrolides or streptogramins	Amoxicillin	161 (39)	276/413 (73)	5			6.8 (1.3)	+ 1.8	37/413 (12%)
			Amoxicillin/clavulanic acid	114 (28)			< RTL	120/413 (3)			
			Macrolids and streptogramins	26 (6)			= RTL	37/413(12)			
			2GC (oral)	1 (1)			> RTL	256/413 (84)			
			No treatment	110 (27)							
	COPD exacerbations (mild) according to recommendations endorsed by AFSSAPS 2011	No treatment	Amoxicillin	161 (39)	110 (27)	0			6.8 (1.3)	+ 6.8	110/413 (27)
			Amoxicillin/clavulanic acid	114 (28)							
			Macrolids and streptogramins	26 (6)			> RTL	303/413 (73)			
			2GC (oral)	1 (1)							
			No treatment	110 (27)							

*According to 2017 French national guidelines endorsed by the French Society of Infectious Diseases (Societé de Pathologie Infectieuse de Langue Française or SPILF); **3GC and TMP-SMX were recommended as second line treatments, ***28/413 (7%) were longer than 10 days.

^1^GASP = group A Streptococcal pharyngitis.

*2GC* 2nd generation cephalosporins, *3GC* 3rd generation cephalosporins, *AFSSAPS* French agency for the sanitary safety of health products or Agence Française de sécurité sanitaire des produits de Santé, *COPD* chronic obstructive pulmonary disease, *GASP* group A Streptococcal pharyngitis, *NA* not available, *RTL* recommended treatment length, *SD* standard deviation, *TMP*-*SMX* trimetoprim-sulfametoxazole, *UTI* urinary tract infections.

**Table 3:** Prescribed treatments (detailed).

Disease		drugs	Level of recommendations*	Prescriptions	Mean treatment lenght	Prolonged treatment	Shortened treatment
				n/tot (%)	Mean (sd)	n/tot (%)	n/tot (%)
Ear-nose-throat	GASP^1^	Amoxicillin	1st line	407/413 (99)	5.9 (0.5)	29/413 (7)	58/413(14)
		Amoxicillin/clavulanic acid	Not recommended	5/413 (1)	6.8 (1.1)	4/413 (1)	1 /413(0)
		Penicillin V	Not recommended	1/413 (0)	7.0 (1.0)	1/413 (0)	0/413 (0)
		Total	–	413/413 (100)	5.9 (0.5)	34/413 (8)	59/413 (14)
	Bacterial maxillary sinusitis	Amoxicillin	1st line	297/413 (72)	6.8 (0.8)	20 /413 (5)	53/413 (14)
		Amoxicillin/clavulanic acid	2nd line	49/413 (12)	7.2 (1.0)	9/413 (2)	3/413 (0)
		Pristinamycin	2nd line	21/413 (5)	4.7 (1.3)	6/413 (2)	0/413 (0)
		3GC	2nd line	21/413 (5)	8.0 (1.7)	3/413 (1)	0/413 (0)
		Other molecules	Not recommended	9/413 (2)	5.8 (2.2)	1/413 (0)	0/413 (0)
		No treatment	Not recommended	37/413 (9)	5.8 (2.2)	1/413(0)	0/413 (0)
		Total	–	413/413 (100)	6.7 (1.0)	39/413 (10)	56/413 (14)
	Bacterial frontal sinusitis	Amoxicillin/clavulanic acid	1st line	328/413 (79)	7.1 (1.1)	50/413 (12)	29/413 (7)
		Amoxicillin	Not recommended	56/413 (14)	6.9 (1.2)	10/413 (2)	15/413 (4)
		Macrolids	Not recommended	10/413 (2)	5.7 (0.4)	3/413 (1)	4/413 (1)
		Fluoroquinolones	2nd line	7/413 (2)	7.8 (0.4)	3/413 (1)	0/413 (0)
		3GC (oral)	2nd line	6/413 (1)	7.4 (1.2)	2/413 (0)	0/413 (0)
		2GC (oral)	2nd line	6/413 (1)	5 (0.9)	0/413 (0)	0/413 (0)
		Total	–	413/413 (100)	7.1 (1.0)	70/413 (17)	48/413 (12)
	Congestive otitis (paediatric)	No treatment	1st line	289/413 (70)	-	-	-
		Amoxicillin	Not recommended	118/413 (29)	5.7 (1.0)	49/413 (12)	-
		3GC (oral)	Not recommended	2/413 (0)	7.0 (0)	2/413 (0)	-
		Fluoroquinolones	Not recommended	2/413 (0)	7.5 (3.5)	1/413(0)	-
		2GC (oral)	Not recommended	1/413 (0)	7.0 (0.0)	0/413 (0)	-
		Amoxicillin/clavulanic acid	Not recommended	1/413 (0)	7.0 (0.0)	0/413 (0)	-
		Total	–	413/413 (100)	5.8 (1.1)	52/413 (13)	-
Urinary	Cystitis (women)	Fosfomycin	1st line	397/413 (96)	1.0(0.0)	0/413 (0)	-
		Pivmecillinam	2nd line	10/413 (2)	5.3 (0.8)	1(0,2)	-
		Nitrofurantoin	2nd line	1/413 (0)	3.8 (2.3)	0/413 (0)	-
		Fluoroquinolones	Not recommended	1/413 (0)	2.0 (1.4)	0/413 (0)	-
		Amoxicillin	Not recommended	1/413 (0)	5.0 (0.0)	0/413 (0)	-
		No treatment	Not recommended	3/413 (1)	14.0 (0.0)	1 (0,2)	-
		Total	–	413/413 (100)	1.1 (1.0)	2 (0,5)	-
	Pyelonephritis (women)	Fluoroquinolones	1st line	311/413 (76)	8.4 (2.4)	110 (29)	13/413 (4)
		3GC (intramuscular/intravenous)	1st line	56/413 (14)	8.9 (2.8)	23 (6)	5/413 (1)
		Amoxicillin	2nd line	22/413 (5)	8.8 (2.1)	1 (0)	9/413 (2)
		3GC (oral)	2nd line	14/413 (3)	9.1 (2.4)	2 (0)	8/413 (2)
		Amoxicillin/clavulanic acid	2nd line	4/413 (1)	7.5 (2.1)	0/413 (0)	3/413 (0)
		Pivmecillinam	Not recommended	4/413 (1)	6.3(1.2)	0/413 (0)	0/413 (0)
		Fosfomycin	Not recommended	1/413 (0)	1.0 (0)	0/413 (0)	0/413 (0)
		Nitrofurantoin	Not recommended	1/413 (0)	8.0 (0)	0/413 (0)	0/413 (0)
		Total	–	413/413 (100)	8.5 (2.4)	136/413 (36)	38/413 (10)
	UTI (men)	Fluoroquinolones	1st line	361/413 (88)	NA	69/413 (17)	53/413 (13)
		3GC (intramuscular)	2nd line	32/413 (8)	NA	3/413 (1)	8/413 (2)
		TMP/SMX	2nd line	10/413 (2)	NA	1/413 (0)	0/413 (0)
		3GC (oral)	Not recommended	5/413 (1)	13.4 (4.9)	0/413 (0)	1/413 (0)
		Amoxicillin	2nd line	3/413 (1)	14 (5.1)	0/413 (0)	0/413 (0)
		Amoxicillin/clavulanic acid	Not recommended	2/413 (0)	NA	1/413(0)	0/413 (0)
		Total	–	413/413 (100)	NA	74/413 (18)	62/413 (15)
Skin	Bacterial superficial skin infections	Amoxicillin	1st line	347/413 (84)	9.5 (1.9)	32/413 (8)	7/413 (2)
		Amoxicillin/clavulanic acid	Not recommended	33/413 (8)	9.8 (2.6)	7/413 (2)	0/413 (0)
		Pristinamycin	2nd line	23/413 (6)	9.8 (2.3)	4/413 (1)	1/413 (0)
		Penicillin M	Not recommended	8/413 (2)	8.9 (2.7)	1/413 (0)	1/413 (0)
		Fusidic acid	Not recommended	2/413 (0)	9.0 (1.4)	1/413 (0)	0/413 (0)
		Total	–	413/413 (100)	9.5 (2.1)	45/413 (11)	9/413 (2)
	Dog bite wounds	Amoxicillin/clavulanic acid	1st line	359/413 (87)	6.1 (1.7)	177/413 (44)	16/413 (4)
		Doxycyclin	2nd line	24/413 (6)	7.0 (1.9)	4/413 (1)	0/413 (0)
		Pristinamycin	2nd line	11/413 (3)	7.1 (1.4)	19/413 (5)	0/413 (0)
		Amoxicillin	Not recommended	5/413 (1)	6.0 (1.8)	8/413 (2)	1/413 (0)
		No treatment	Not recommended	14/413 (3)	6.0 (1.8)	8/413 (2)	1/413 (0)
		Total	–	413/413 (100)	6.2 (1.7)	208/413 (52)	17/413 (4)
Pulmonary	Community-acquired pneumonia (≥ 65 year old)	Amoxicillin/clavulanic acid	1st line	170/413 (41)	7.8 (1.7)	54/413 (13)	5/413 (1)
		Amoxicillin	Not recommended	229/413 (56)	8.2 (1.8)	90/413 (22)	10/413 (2)
		Levo	1st line	5/413 (1)	9.2 (3.4)	3/413 (1)	1/413 (0)
		Pristi	Not recommended	4/413 (1)	8.5 (1.0)	4/413 (1)	3/413 (0)
		3GC (intravenous)	1st line	4/413 (1)	5.0 (2.7)	1/413 (1)	3/413 (0)
		Macrolids	2nd line	1/413 (0)	5.0 (0.0)	0/413 (0)	1/413 (0)
		Total	–	413/413 (100)	8.0 (1.8)	152/413 (37)	23/413 (6)
	COPD exacerbations (mild)	No treatment	**	110 (27)	–	–	–
		Amoxicillin	1st line	161 (39)	6.9(1.0)	144/413 (48)	0/413 (0)
		Amoxicillin/clavulanic acid	1st line	114 (28)	7.1 (1.3)	100/413 (34)	0/413 (0)
		Macrolids/Pristinamycin	1st line	26 (6)	5.1 (1.3)	6/413 (2)	10/413 (2)
		2GC	Not recommended	1 (1)	7.1 (1.2)	1/413 (0)	0/413 (0)
		Total	–	413/413 (1)	6.8 (1.3)	251/413 (84)	10/413 (2)

*According to 2017 French national guidelines endorsed by the French society of infectious diseases (societé de pathologie infectieuse de langue Française or SPILF), **Recommended by the AFSSAPS (French agency for the sanitary safety of health products or agence Française de sécurité sanitaire des produits de santé).

^1^GASP = group A Streptococcal pharyngitis.

*2GC* 2nd generation cephalosporins, *3GC* 3rd generation cephalosporins, *COPD* chronic obstructive pulmonary disease, *GASP* group A Streptococcal pharyngitis, *NA* not available, *SD* standard deviation, *TMP*-*SMX* trimetoprim-sulfametoxazole, *UTI* urinary tract infections.

Diseases with a correct response rate higher or equal to 70% included cystitis, GASP, paediatric congestive otitis, and bacterial superficial skin infections. Paediatric congestive otitis (with a viral cause) was incorrectly prescribed antibiotics by 123/413 (30%) physicians.

Diseases with a correct response rate between 51 and 69% included bacterial frontal sinusitis, bacterial maxillary sinusitis, and UTIs. For bacterial sinusitis (both maxillary and frontal), the combined correct response rate was approximately 60%.

Diseases with a correct response rate lower or equal to 50% included pyelonephritis, dog bite wounds, CAP in patients aged 65 and above, and COPD exacerbations. The combined correct response rate for these diseases was 43%.

Pyelonephritis in women was accurately treated in 48% of cases, despite 30% of the prescriptions suggesting a treatment duration longer (10 to 14 days) than guidelines (7 days).

For prophylactic management of dog bite wounds, only 41% of physicians adhered to the recommended treatment duration. Treatment durations frequently exceeded guidelines, with an average of 6.1 days (+ 1.1 days) for the first-line antibiotic (Amoxicillin/clavulanic acid), 7.0 and 7.2 days (+ 2 and + 2.2 days) for the second-line antibiotics (Doxycycline and Pristinamycin). In some instances, the treatment extended to as long as 10 days in 7% of prescriptions.

As for COPD exacerbations, approximately 1/3 of the physicians adhered to the AFFSAPS recommendations, opting not to prescribe antibiotics, while 2/3 of the physicians prescribed antibiotics, in alignment with the SPILF guidelines. Within this context, an appropriate answer in terms of both drug and treatment duration was observed in 37/413 (12%) responses.

For the management of community-acquired pneumonia in elderly patients (aged ≥ 65 years), a mere one-third (27%) of physiciens adhered to the guidelines regarding the first-line antibiotic (amoxicillin/clavulanic acid) and treatment duration (7 days).

Factors associated with the propensity for overtreatment included: age over40 years, practising in rural settings, and holding a hospital-based position, as detailed in Table 4.

**Table 4:** Over-prescription’s risk factors.

Characteristics	Duration score			Treatment score		
	≥ 75% quartile	< 75% quartile	p-value	≥ 75% quartile	< 75% quartile	p-value
	n = 88	n = 325		n = 89	n = 324	
Male gender, n [%]	22 [25]	100 [31]	0.1	23 [25]	100 [31]	0.2
Age > 40 years, n [%]	22 [25]	35 [11]	0.001	18 [20]	39 [12]	0.03
Country setting, n [%]	19 [22]	65 [20]	0.5	20 [22]	67 ^21^	0.04
Hospital job, n [%]	16 [18]	32 [10]	0.3	20 [22]	29 [9]	< 0.001
Independent job, n [%]	19 [22]	57 [18]	0.6	33 [39]	55 [17]	0.3

## Discussion

This study assessed the knowledge level of GPs for recommended treatments and the factors associated with the over-prescription of antibiotics for community-acquired infections. We found that physicians aged over 40 years (p = 0.001) were associated with antibiotic over-prescription. This finding is consistent with previous data on antibiotic consumption in France, which suggests an association between antibiotic over-prescription and the age of the practitioner^11–13^. The inclination for over-treatment among hospital doctors (p < 0.001) is more challenging to interpret. One hypothesis is that their responses could be influenced by their routine exposure to critically ill patients, often requiring broad spectrum antibiotic treatments.

Heterogeneous levels of knowledge were observed according to the different diagnoses: low (≤ 50% of correct answers), mild (51–69% of correct answers) and high (≥ 70% of correct answers).

Prescription of antibiotic prophylaxis following an animal (dog) bite is a standard practice endorsed by several national guidelines, including French guidelines^2,14^. In our study, the duration of antibiotic prophylaxis for dog bite wounds exceeded the recommended duration (mean value 6.2 days vs 5 days). This discrepancy might be attributed to the French guidelines, which allow for an extension of treatment duration up to 10 days when signs of local or general infection are presents^2^. Yet, the likelihood of infection post-animal bites vary based on the context and the animal species involved. In particular, cat bite wounds tend to be more frequently infected than dog bite wounds^15^. Moreover, surgical intervention plays a significant role in reducing wound infection. In some instances, surgical repair proves as effective as antibiotic prophylaxis in preventing wound infection post-animal bites^16,17^, Further research is required in order to determine the optimal management strategy for animal bites while reducing antibiotic over-prescription.

Our research showed that over 30% of practitioners recommended a longer antibiotic treatment (10 to 14 days) than guidelines (7 days) for treating pyelonephritis in women. Such findings are of significant concern, given that fluoroquinolones (first-line antibiotics) are broad-spectrum antibiotics, which play a pivotal role in the emergence of bacterial resistance. The problem also arises, albeit to a lesser extent, with second line antibiotics (third generation cephalosporins). As part of ongoing efforts to fight antibiotic resistance, new French guidelines for the management of urinary tract infections were published in 2021. In these recommendations, fluoroquinolones have been downgraded to third-line drugs, with trimethoprim-sulfametoxazole emerging as the preferred first-line antibiotic^18^. Still, there’s room for improvement, especially concerning the antibiotic duration. Indeed, shorter treatment durations (5 days) for simple acute pyelonephritis are recommended by guidelines endorsed by the European Association of Urology^19^.

UTIs in males were generally managed appropriately in terms of antibiotic selection, but results were poor when duration of antibiotic treatment was considered. Indeed, 19% of prescriptions exceeded the recommended duration (14 days). The optimal duration for antibiotic treatment for UTIs in males remains controversial. The various presentations of UTIs in males (cystitis, pyelonephritis and prostatitis) are often difficult to define, especially in a community-based setting^20^.

In this study, only one-third (27%) of practitioners adhered to the guidelines regarding the choice of first line drug (amoxicillin/clavulanic acid) and duration (7 days) for managing CAP in individuals aged 65 and above. A potential confounding factor may be the older guidelines endorsed by SPILF and AFFSAPS in 2010, which recommended the use of amoxicillin for 7–14 days for both adults and the elderly^9^. Furthermore, prevailing medical practices might be influenced by other factors, either intrinsic (e.g. practitioner’s experience and potential gaps in ongoing training) or extrinsic (e.g. patients’ demands and pharmaceutical representatives), as corroborated by prior research^21,22^. Our findings emphasize the lack of information and highlight the need for improving the distribution of endorsed guidelines. New French guidelines are currently in progress considering the results of a recent trial which demonstrated the non-inferiority of 5-day treatment *versus* the standard treatment (7 days) for patients with a CAP and a favourable evolution^23^. However, improvements are required for the choice of molecules as well. Indeed, France ranks as one of the top consumers of extended spectrum penicillins ± beta lactamase inhibitors within the European Union^24^. One of the main indication for extended spectrum penicillins in France is pneumonia, while narrow spectrum penicillins are preferred in other European countries^8,25^. Moreover, guideline recommendations are predominantly shaped by hospital-based specialists who face routinely severe pneumonia cases requiring an urgent life-saving antibiotic treatment. Consequently, broad-spectrum molecules (e.g. beta-lactam + beta-lactamase inhibitor) are usually prescribed in hospital settings. Conversely, narrow-spectrum antibiotics are more fitting for non-severe outpatient cases, aligning with guidelines from other European countries. This strategy is similar to the United Kingdom’s approach, which favours amoxicillin as first-line treatment^26^. The variations in national guidelines can be attributed to the level of knowledge of CAP microbiology. Further research is necessary to improve knowledge of pneumonia epidemiology in France with the objective to minimize the use of broad spectrum penicillins.

This study showed moderate findings related to the treatment of ear-nose-throat infections. For bacterial sinusitis, the combined accuracy rate (for both maxillary and frontal) was almost 60%. Moreover, an inclination to prescribe a treatment exceeding the recommended duration (7 days) was observed for second line drugs (fluoroquinolones, third generation cephalosporins). For congestive otitis in children, 30% of practitioners prescribed antibiotic therapy, even though no antibiotic treatment is recommended according to SPILF guidelines, as the majority of congestive otitis cases are viral in origin^2^. It is possible that many practitioners would be misled by other guidelines which recommended amoxicillin as first line treatment of otitis media^27^. These findings are in line with those from others studies, and emphasize the necessity of improving educational interventions among GPs^28^. Indeed, antimicrobial stewardship programs can focus on situations where “antibiotic treatment is contraindicated or not beneficial”^29^. The decrease of antibiotic prescriptions after antimicrobial stewardship programs was demonstrated in case of viral infections in community^30^.

Exacerbations of COPD are common in a GP’s daily practice. As the general population ages, with the consequent increase in comorbidities, its burden is expected to grow in the following years^31^. While antibiotics are fundamental to treating COPD exacerbations, their appropriate administration in instances of severe, albeit non- life-threatening exacerbations remains a subject of debate. The Global Initiative for Chronic Obstructive Lung Disease (GOLD) guidelines suggest using oral antibiotics in the presence of signs of infections, but the best strategy to detect infection remains controversial. The most prevalent method to evaluate infection risk during a COPD exacerbation is the evaluation of sputum purulence, though its use in a GP’s clinical practice is limited by its interpretive subjectivity. Additionally, sputum culture isn’t practical in community settings. Similarly, the use of biomarkers of infection during COPD exacerbation (C-reactive protein, procalcitonin) remains controversial, with research yielding inconclusive outcomes^32^. Moreover, an important confounding factor is the existence of divergent guidelines. In France, AFSSAPS 2011 guidelines did not recommend any antibiotic treatment, while SPILF 2017 guidelines recommended a 5-day antibiotic course^2,8^. Given this lack of clear consensus, the management of COPD exacerbations is often based more on the practitioner’s clinical experience rather than established guidelines^33,34^. Thus, the prescription of an antibiotic treatment during a COPD exacerbation frequently presents a conundrum for GPs. In this context of confusion, harmonization of guidelines is advisable.

For cystitis in women, GASP, and bacterial superficial skin infections, no criticisms were highlighted by this study.

This study presented several limitations: (i) the survey was diffused principally through internet and social media. Consequently, a younger population was selected (31 years old for this study vs 57.1 years old for French GP populations). Because age is a cause of over-treatment, our study likely underestimates prescription times; (ii) the absence of some data in the clinical cases included in the survey (duration of symptoms, re-evaluation after 48 h from the beginning of the antibiotic therapy) reduced the evaluation of treatment duration; (iii) The survey was designed to be completed by each prescriber in less than 15 min. For this reason, some frequent community-acquired infectious diseases were excluded, such as: cystitis in pregnant woman, acute diarrhoea, otitis media in children less than 2 years old; (iv) This study was based on a web questionnaire. Answers could attest the global knowledge of GPs rather than their attitude in daily clinical practice.

## Conclusions

This study presented the answers to a Web based survey in a French region (*Occitanie*). It analysed the knowledge of 413 GPs about official guidelines concerning 11 infectious diseases which are routinely managed in GP’s office: GASP, bacterial maxillary sinusitis, bacterial frontal sinusitis, congestive otitis in children, cystitis in women, pyelonephritis in women, UTI in men, bacterial superficial skin infections, dog bite wounds, CAP in ≥ 65-year-old patients, COPD mild exacerbation.

According to this survey, knowledge of treatment durations is satisfactory with 60% of recommendations being met. However, heterogeneous levels were observed according to different diseases: (i) diseases with low level of knowledge (≤ 50% of correct answers) were pyelonephritis, dog bite wounds and CAP in patients ≥ 65-year-old.; (ii) diseases with mild level of knowledge (51–69% of correct answers) were bacterial frontal sinusitis, bacterial maxillary sinusitis and UTI; (iii) diseases with high level of knowledge (≥ 70% of correct answers) were cystitis, group A streptococcal pharyngitis and bacterial superficial skin infections. Moreover, the study identified age > 40-year-old, country setting and hospital job as factors associated with the risk of overtreatment.

Results of this study shows that rate of adherence is high when recommendations are clear while it falls when different guidelines existed or are not clear. According to data presented in this article, there is a limited margin of progression for the proper use of antibiotics when guidelines are clear. It is necessary to provide simplified and updated guidelines through channels accessible to GPs.

## Data Availability

The datasets used and/or analysed during the current study are available from the corresponding author on reasonable request.

## Abbreviations

AFSSAPS: French agency for the Sanitary Safety of Health Products (*Agence Française de Sécurité Sanitaire des Produits de Santé*)
CAP: Community-acquired pneumonia
COPD: Chronic obstructive pulmonary disease
DDD: Daily defined dose
ENT: Ear-nose-throat
GASP: Group A streptococcal pharyngitis
GOLD: Global initiative for chronic obstructive lung disease
GP: General practitioner
SPILF: French Society of Infectious Diseases (*Societé de Pathologie Infectieuse de Langue Française*)
UTI: Urinary tract infection
